# Variations in T-helper 17 and Regulatory T Cells during The Menstrual Cycle in Peripheral Blood of Women with Recurrent Spontaneous Abortion

**Published:** 2014-03-09

**Authors:** Nasrin Sereshki, Marjan Gharagozloo, Vajihe Ostadi, Ataollah Ghahiri, Mohammad Ali Roghaei, Ferdos Mehrabian, Alireza Andalib Andalib, Akbar Hassanzadeh, Habibolla Hosseini, Abbas Rezaei

**Affiliations:** 1 Department of Immunology, School of Medicine, Isfahan University of Medical Sciences, Isfahan, Iran; 2 Department of Obstetrics and Gynecology, Al-Zahra Hospital, Isfahan University of Medical Sciences, Isfahan, Iran; 3 School of Public Health, Isfahan University of Medical Sciences, Isfahan, Iran; 4 Isfahan University of Medical Sciences, Isfahan, Iran

**Keywords:** Regulatory T Cells, T Helper 17, Menstrual Cycle, Pregnancy

## Abstract

**Background:**

Disorders in immune system regulation may result in pregnancy abnormalities such as recurrent spontaneous abortion (RSA). This study aims to determine the ratio of regulatory T (Treg) and T helper (Th) 17 cells in unexplained RSA (URSA) women during proliferative and secretory phases of their menstrual cycles compared to healthy non-pregnant women.

**Materials and Methods:**

In this case control study, 25 women with URSA and 35 healthy, non-pregnant women were enrolled. The percentage of Th17 and Treg cells in participants peripheral blood were determined by flow cytometry.

**Results:**

The percentage of Th17 cells and their related cytokines in serum (IL-17A) were higher in the proliferative and secretory phases of the menstrual cycles of URSA women compared to the control women. However, a lower percentage of Treg cells and their related cytokines in serum, transforming growth factor (TGF) β1 and interleukin (IL)-10 were detected in the proliferative but not the secretory phase of the URSA group. The ratio of Th17/CD4+ Treg was higher in the URSA group than the control group. We observed an increased ratio of Th17/CD4+ Treg during the proliferative and secretory phases in URSA women.

**Conclusion:**

The imbalance between Th17 and Treg cells during the proliferative phase of menstrual cycles in the URSA group may be considered a cause for spontaneous abortion.

## Introduction

The combination of numerous factors result in a successful pregnancy, of which cells and molecules of the immune system may be the most important. Disturbances in regulating these cells and molecules can result in an aberrant pregnancy such as recurrent spontaneous abortion (RSA). RSA is a common problem among couples and is defined as the occurrence of three or more clinically detectable pregnancy losses that usually occur prior to 20 weeks of gestation (1). The multiple causes of RSA include uterine anatomical defects, uterine infections, chromosomal aberrations, hormonal disorders, hematological problems and immunological abnormalities (2).

Immunological dysfunctions may cause impaired maternal immune tolerance to the fetus and result in fetal rejection. Aberrant expression of human leukocyte antigen (HLA), autoimmune diseases and autoantibodies, T helper1/T helper2 (Th1/Th2) imbalance, in addition to varied functions of uterine natural killer cells are examples of immunological dysfunctions (3). One of the most important immunological factors that plays a primary role in controlling the maternal immune response and results in a successful pregnancy are regulatory T (Treg) cells (4). Treg cells suppress excessive immune response of other cells and maintain tolerance to self-antigens. Several types of Treg cells have been identified and include CD8+ Treg cells, induced interleukin-10 (IL-10)-producing Treg cells (Tr1), TGF-β-producing Treg cells (Th3), and CD4+ CD25+ FOXP3+ Treg cells (5).

CD4+ CD25+ FOXP3+ Treg cells are one of the best-characterized subsets of immune regulatory cells. The two forms of these cells are natural Treg cells which are formed in the thymus and inducible Treg cells which are formed in the periphery during antigen-specific stimulation (6). Suppressive activity of Treg cells occurs by at least two mechanisms: i. cell-cell contact through expression of inhibiting molecules such as programmed death-1 (PD-1) or cytotoxic T-lymphocyte antigen (CTLA-4) or ii. via secretion of immunosuppressive cytokines such as transforming growth factor beta (TGF-β) and IL-10 (7, 8). 

Human CD8+ Treg cells have been studied in less detail. Regulatory properties of these cells are similar to CD4+ FOXP3+ Treg cells (9). Although they express prostaglandin E2 (PGE2), IL-10 and TGF-β, their suppressive function appears to be cell contact-dependent. These cells recognize class Ib protein HLA-E which is expressed by most human tissues and cell lines, but at lower levels than MHC class Ia antigens (10, 11). Human trophoblast cells express HLA-E that plays an important role in protection of the fetus from maternal rejection by natural killer (NK) cells (12).

Th17 cells have been described as a subset of Th cells (CD4+) which play a major role in induction of inflammation by producing proinflammatory cytokines such as IL-17A, IL-17F, IL-22, IL-6, TNF-α, and matrix metalloproteinase. Recent data have shown a pathogenic effect of these cells in autoimmunity, transplant rejection and other diseases (13, 14). Several studies have shown the balance between Treg cells and TH17 cells under normal and pathologic conditions (15-17).

To the best of our knowledge there are few studies on the role of Th17 and Treg cells in unexplained RSA (URSA). This study evaluates and compares the percentage and ratio of Th17 and Treg cells in peripheral blood of women with URSA to healthy (proven fertile) non-pregnant women. We have studied the frequency and ratio of Th17/Treg cells in URSA women who were at least three-months after their last abortion. We propose that URSA may be the result of an irregularity in these cells and their balance before embryo implantation or pregnancy.

## Materilas and Methods

### Subjects

In this case control study, a total of 25 URSA women with a mean age of 29.45 years (range: 21- 43 years) who had at least three consecutive first trimester abortions were enrolled. The diagnosis of URSA was made after excluding any definite caus- es such as abnormalities of the uterus or cervix, chromosomal abnormality, infection, endocrine and metabolic diseases, congenital thrombophilias and autoimmune disease. All male partners had normal semen status, according to criteria from the World Health Organization. The control group comprised 35 non-pregnant healthy women with a mean age of 30.5 years (range: 22-42 years) who had at least one successful pregnancy without any disease. Control group women had no history of any still birth, preterm and post-term labor, ectopic pregnancy, preeclampsia and abnormal pregnancy. All participants were at least three months from their last abortion or pregnancy. Blood samples were taken from both groups. After sampling, subjects were assigned to either of two groups, secretory or proliferative phase. These subjects had regular menstrual cycles of 26-31 days. Women in the last 14 days of their menstrual cycles comprised the secretory group, whereas those prior to the last 14 days of their menstrual cycles were considered to be the proliferative group. Women with menses were removed from the study. Clinical characteristics of URSA and control group women are summarized in table 1. 

**Table 1:** Clinical characteristics of subjects


Clinical characteristics	URSA	Normal

Age (Y)	29.9 ± 5.7	30.3 ± 5.8
Proliferative phase (Day of menstrual cycle)	10.0 ± 1.2 (n=13)	10.8 ± 2.1(n=15)
Secretory phase (Day of menstrual cycle)	20.7 ± 4.7 (n=12)	21.1 ± 3.4 (n=20)
Number of abortions	3.7 ± 1.2	0.0 ± 0.0


Results were expressed as the mean ± SD. N; Number of subjects in the phases and URSA; Unexplained recurrent spontaneous abortion.

### Blood samples

A total of 8 ml heparinized venous blood and 2 ml without anticoagulant were taken for the ELISA test from the case and control groups. Sera were separated using centrifugation and stored at -80˚C until use.

### Isolation of peripheral blood mononuclear cells

Peripheral blood mononuclear cells (PBMC) were separated from freshly isolated heparinized venous blood by centrifugation on a Ficoll-Hypaque (Lymphoprep, Sigma, USA) density gradient. Cells at the interface were harvested, washed twice, and resuspended in phosphate buffered saline (PBS). Viable cells were counted by the trypan blue staining method. More than 95% of the cells were viable.

### Flow cytometric analysis 

For analysis of Th17 cells, PBMC (2×10^6^ cells/ml) were suspended in complete culture medium that contained RPMI 1640 with L-glutamine, penicillin (100 U/ml), streptomycin (10 mg/ml) and 10% fetal bovine serum (FBS). This cell suspension (2×10^6^ cells/ml) was transferred to the 24-well plates. Phorbol 12-myristate 13-acetate (PMA, 150 ng/ml) and ionomycin (1 μM) were added to each well in the presence of monensin (500 ng/ ml) for 12 hours (all purchased from Sigma, USA). Then, cells were incubated at 37˚C in a humidified 5% CO_2_ atmosphere. After 12 hours, cells were washed with PBS at 1500 rpm for 5 minutes. After stimulation of PBMC *in vitro*, the cells were incubated with fluorescein isothiocyanate (FITC) anti-human CD4 at 4˚C for 30 minutes. After surface staining, the cells were fixed and permeabilized with BD Cytofix/Cytoperm solution and stained with phycoerythrin (PE) anti-human IL-17A.

For CD4+ Treg staining cell, PBMC (2×10^6^ cells/ml) were incubated with PerCP anti-human CD4. In order to stain CD8+ Treg cells, PBMC were incubated with FITC anti-human CD8. After surface staining, the cells were fixed and permeabilized with BD Cytofix/ Cytoperm solution and stained with PE antihuman FOXP3.

**Fig 1:**
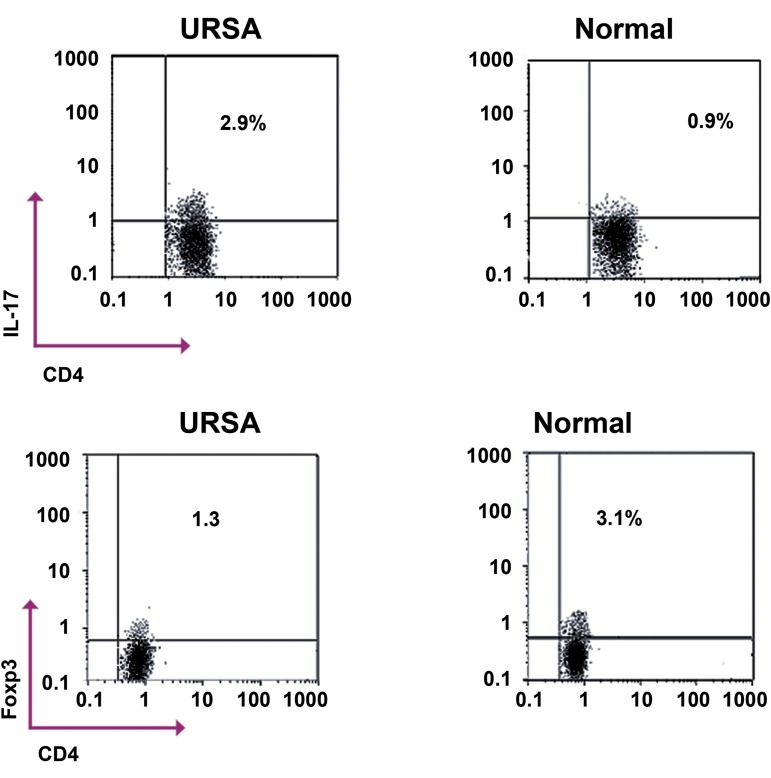
Representative flow cytometry dot plots of TH17 (CD4+ IL-17A+ ) and Treg (CD4+ FOXP3+ ) in unexplained recurrent spontaneous abortion (URSA) and healthy, normal women. Plots shown were gated on CD4+ lymphocytes. The percentage of cells falling into the respective quadrants is indicated in each plot. *; Considered significant compared with the control group according to independent sample t test and p=0.001

Isotype controls were given to enable correct compensation and antibody specificity onfirmation. All antibodies were purchased from BD Pharmingen, USA. As seen in figure 1, the percentages of Th17 or Treg cells were determined using a flow cytometer (Partec, Germany). A total of 50000 cells were analyzed for detection of Th17 and Treg cells. We used Flowmax software for data analyses.

### ELISA

The serum concentrations of IL-17A, TGF-β1 and IL-10 were measured using an ELISA kit in accordance with the manufacturer’s protocol (Boster Biological Technology Co., Wuhan, China). The ELISA kits had a sensitivity of 1 pg/ml for TGFβ and IL-17 and 0.5 pg/ ml for IL-10. All samples were measured in duplicate.

### Statistical analyses

Analyses with Levene’s test for Treg cells and Th17 cells showed equal variances. Therefore the data distribution was normal. We used the independent sample t test to analyze the significance of difference in the mean percentage of Th17, Treg cells (both CD8+ and CD4+ ), and the Th17/CD4+ Treg ratio between the case and control groups. Levene’s test for cytokine analyses showed unequal variances (not normal distribution). Therefore we used non-parametric statistics. The Mann-Whitney U test was used to compare cytokines in the proliferative and secretory phases of URSA and with control group women. SPSS software was used.

### Ethical considerations

The protocol for this study was approved by the Ethics Committee of Isfahan University of Medical Sciences (Isfahan, Iran). Informed consent was obtained from all subjects who participated in this study.

## Results

### Th17 frequencies in peripheral blood

Table 2 shows that the percentage of Th17 cells (CD4+ IL17+ ) was significantly higher in women with URSA than healthy non-pregnant women. Results also revealed a higher percentage of TH17 cells in the proliferative and secretory phases of menstrual cycles in women with URSA compared to healthy non-pregnant women.

**Table 2:** Percentages of CD4+ Treg, CD8+ Treg, and CD4+ Th17 cells in peripheral blood from unexplained recurrent spon- taneous abortion (URSA) and healthy, normal women in the proliferative and secretory phases of the menstrual cycle and without considering phase


	Proliferative phase			Secretory phase			Without considering phase		
Cell subsets	URSA	Normal	P value	URSA	Normal	P value	URSA	Normal	P value

CD4^+^IL-17A^+^	1.6 ± 1.5*	0.6 ± 0.4	0.004	2.0 ± 1.3*	0.5 ± 0.4	0.001	1.8± 1.4*	0.6 ± 0.4	0.001
CD4^+^FOXP3^+^	0.9 ± 1.1*	2.3 ± 1.7	0.010	1.3 ± 1.2	1.4 ± 1.5	0.410	1.1±1.1*	1.9 ± 1.7	0.030
CD8^+^FOXP3^+^	0.2 ± 0.1	0.2 ± 0.1	0.110	0.1 ± 0.1	0.3 ± 0.3	0.09	0.1± 0.1*	0.3 ± 0.2	0.040


Results are expressed as mean ± SD. Values for cells are expressed as percentages. *; Considered significant compared with the control group (p≤0.05).

### Treg frequencies in peripheral blood

Results showed a significantly lower percentage of CD4+ FOXP3+ Treg cells in the URSA group compared to the control group. However, the decreased percentage of Treg was detected in the proliferative phase but not in the secretory phase (Table 2). The percentage of CD8+ FOXP3+ Treg was lower in the URSA group compared to the control group. There was no significant change in percentage of CD8+ FOXP3+ Treg in the proliferative and secretory phases of menstrual cycles in women with URSA compared to healthy non-pregnant women. 

### Ratio of Th17/CD4+ Treg in peripheral blood

**Fig 2:**
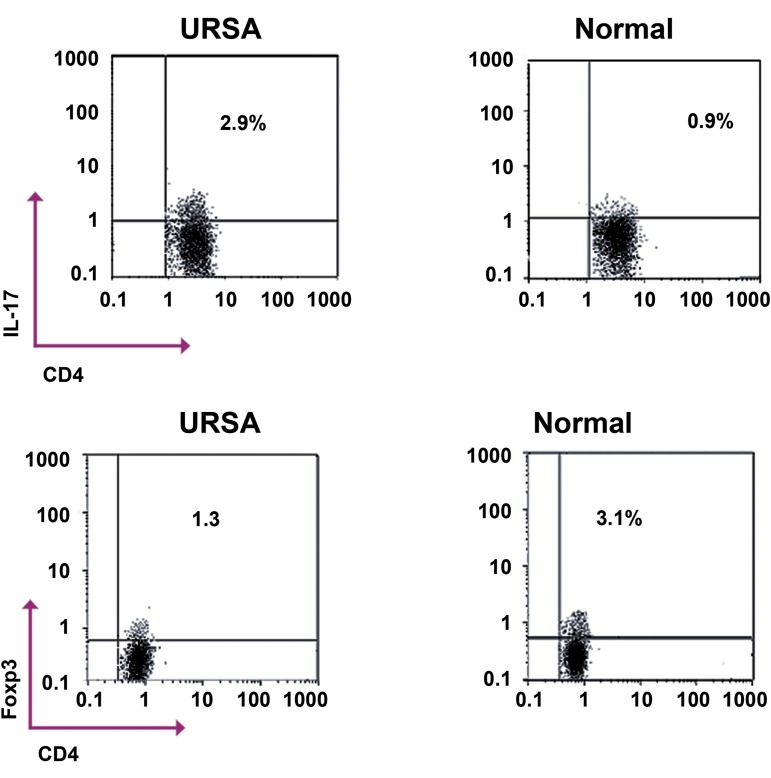
TH17/CD4+ Treg ratio in RSA and normal women. *; Considered significant in comparison with control (used sta- tistical test is independent sample t test and p value=0.001).

**Fig 3:**
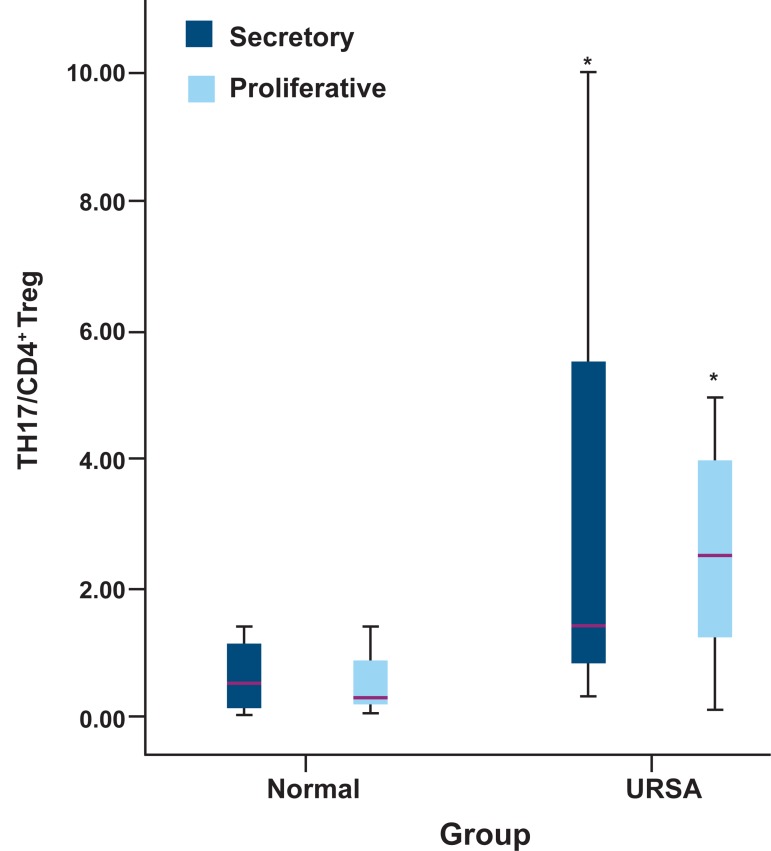
TH17/CD4+ Treg ratio in proliferative and secretory phase in RSA and normalwomen. *; Considered significant in comparison with control (the used statistical test is independent sample t test and p value in secretory phase=0.008 and in proliferative phase, p value=0.06).

Figure 2 shows a higher ratio of Th17/CD4+ Treg in the URSA group compared with the control group. An increased ratio of Th17/CD4+ Treg cells was observed in the proliferative and secretory phases of URSA patients (Fig 3).

### Cytokine concentrations in serum

According to table 3 the IL-17 concentrations in sera of women with URSA were significantly higher compared to the control group. However, the levels of TGF-β1 and IL-10 in women with URSA were significantly lower than those of the control group. Results also revealed a higher IL- 17 concentration in the proliferative and secretory phases of the menstrual cycle. TGF-β1 and IL- 10 concentrations were lower in the proliferative phase in women with URSA compared to the con- trol group (Table 3). 

**Table 3:** Concentration of cytokines in serum from unexplained recurrent spontaneous abortion (URSA) and healthy, normal women in the proliferative and secretory phases of menstrual cycles


	Proliferative phase			Secretory phase			Without considering phase		
Cytokine (pg/ml)	URSA	Normal	P value	URSA	Normal	P value	URSA	Normal	P value

IL-17A	13.5 ± 1.2*	12.4 ± 1.6	0.030	14.6 ± 1.8*	12.4 ± 1.1	0.004	14.0 ± 1.5*	12.4 ± 1.4	0.032
TGF-β1	20.4 ± 13.5*	50.4 ± 8.9	0.002	55.6 ± 16.2	51.6 ± 22.1	0.090	36.6 ± 23.0*	50.8 ± 7.0	0.002
IL-10	9.3 ± 2.3*	33.0 ± 12.5	0.040	14.9 ± 8.7	15.9 ± 5.8	0.090	11.9 ± 6.6*	26.2 ± 13.3	0.040


Results are expressed as mean ± SD. *; Considered significant compared with the control group (p≤0.05).

## Discussion

In this study, we evaluated the percentage and ratio of TH17 and Treg cells in peripheral blood of women with URSA and compared them with healthy (proven fertile) non-pregnant women. Results of the present study showed a significantly lower percentage and number of Treg cells (CD4+ Foxp3+ and CD8+ FOXP3+) in the peripheral blood of URSA women compared to the control group. Sasaki et al. showed CD4+ CD25 cells in women with spontaneous abortion were equal with nonpregnant women (18). This might be related to differences between the numbers of cases in the RSA group. In the current study we used CD4 and foxP3 to detect Treg cells. Yang et al. showed lower levels of CD4+ CD25bright Treg cells in the peripheral blood of URSA women (1.55 ± 0.77% vs. 2.65 ± 1.10%) which was consistent with our study (19). 

The results of the current study showed a low level of CD4+ FOXP3+ Treg cells in peripheral blood of URSA women in the proliferative or follicular phase of the menstrual cycle compared to the same phase in non-pregnant women. However, no significant change in the number of Treg cells was observed in the secretory or luteal phase (Table 2). A study in peripheral blood of fertile non-pregnant women detected an expansion of Treg cells in the late follicular phase of the menstrual cycle that was followed by a dramatic decrease in Treg numbers in the secretory phase (20). In the late follicular phase, the uterus is ready to accept the embryo. Therefore, a decreased inflammatory response is necessary for uterus preparation (21). Also, the decrease of Treg cells in the secretory phase might assist with implantation, which is an inflammatory process. As a result, the reduced number of Treg cells in the proliferative phase might be the cause for inflammation and subsequent embryo rejection in URSA women.

There are a few studies on the role of CD8+ FOXP3+ Treg cells in pregnancy (22). The present study has shown significantly decreased CD8+ FOXP3+ Treg cells in peripheral blood of URSA women compared to the control group. A recently published study has stated that CD8+ Treg cells have the ability to limit effector T-cell responses in an 'unconventional' major histocompatibility complex (MHC) class Ib-restricted manner. It has been reported that CD8+ Treg cells play a main role in restoring immune homeostasis (23). Therefore reduced frequency of CD8+ Treg cells in URSA women may be related to a change in immunologic homeostatic mechanisms to inhibit fetus rejection.

Treg cells exert part of their function by producing immunoregulatory cytokines such as TGF-β1 and IL-10 (7). Our study has shown a statistically significant reduction in serum levels of cytokines related to Treg cells in URSA women during the proliferative phase. It has been shown that TGF-β1 assists with ovulation, implantation, trophoblast differentiation, immunoregulation at the maternal-fetal interface and in angiogenesis (24). IL-10 is known to be an effective immune-regulating cytokine and inhibitor of inflammatory cytokine synthesis. Several studies have shown that IL-10 controls inflammatory processes in pregnancy (25) and therefore any change in its level may cause an aberrant pregnancy.

This study demonstrated that the percentage and number of Th17 cells (CD4+ IL-17A+) af- ter stimulation of PBMC *in vitro* was higher in URSA women compared to the control group. Lee et al. (26) showed an increasing number of Th17 cells in URSA women (2.2 ± 1.1) compared to controls (1.8 ± 0.5, p=0.021) that agreed with our results. Another studies performed on URSA women at the time of abortion compared to women with elective abortion demonstrated increased frequency of Th17 cells in URSA women (27-29). The present study also demonstrated increased frequency of Th17 cells and elevated levels of IL-17A in peripheral blood of URSA women during both the proliferative and secretory phases of their menstrual cycles. Th17 cells mainly exert their function by means of secreting IL-17A (13). IL-17A is an inflammatory cytokine (30). Therefore increased levels of this cytokine may lead to tissue inflammation and fetus rejection. 

Studies have demonstrated the elevation of Th17 cells in acute tissue rejection (14, 31). Considering the fetus as an allograft (32), Th17 cells may induce rejection of the fetus by producing a variety of pro-inflammatory cytokines that include IL- 17A, IL-17F, IL-21, IL-22, IL-6 and TNFα (13). It has been reported that Th17 can reciprocally convert into Th1,Th2, and Treg cells (13). Recent studies have shown the presence of cells that express IL17/FOXP3 (33) and IFNγ/IL17 (34). These cells are probably used as transient phenotype when the cells convert to each other. This plasticity is under the effect of the cytokine milieu. Since a Th1/Th2 imbalance can trigger an abortion (35), it can be concluded that Th17 conversion into other helper T cell subpopulations probably plays a role in Th1/ Th2 imbalance in RSA

A recently published study on JEG-3 human choriocarcinoma cells demonstrated that IL-17 increased progesterone secretion by JEG-3 cells (35). This suggested that Th17 might be useful for a successful pregnancy. It has been shown that progesterone leads to progesterone production induced blocking factor (PIBF) in pregnancy which favors protection of the fetus (36). In conjunction, these observations suggest that Th17, at its physiologic level, may be crucial for a successful pregnancy. However, elevations of Th17 may cause increased inflammation and disturb the Th1/Th2 balance, leading to RSA. Further studies are still required to determine the role of Th17 cells and Th17/CD4+ Treg cell balance in implantation and pregnancy.

Results of the present study also showed an increased ratio of Th17/CD4+ Treg cells in periph- eral blood of URSA women compared to the control group. This finding was consistent with recent studies that reported increased ratios of Th17/ CD4+ Treg in URSA women (27, 37). The present study showed an increased Th17/CD4+ Treg ratio in the proliferative and secretory phases of URSA women. The Th17/CD4+ Treg balance can be essential for maternal tolerance of the conceptus, hence a reduced level of Treg during the late proliferative phase may correlate with elevations of Th17 in URSA women. This may lead to disrup- tions in the Th17/CD4+ Treg balance and failure of implantation.

It seems reasonable to conclude that URSA and infertility may result from failure of pre-implantation immune mechanisms. It has been shown that elevation of Treg cells before implantation may be caused by processing and presentation of paternal alloantigen that is present in seminal fluid by dendritic cells in endometrial and cervical tissues (20). This, together with the high levels of TGFβ and prostaglandin E in seminal fluid may therefore activate Ag-dependent CD4+ Treg and CD8+ regu- latory cells prior to conceptus antigen encounter with maternal tissue (5, 20). Therefore, an abnormality in recognition of paternal alloantigens may be a possible cause for decreased Treg cells and consequently RSA.

## Conclusion

Th17 cells at their physiologic level may be necessary for successful implantation, whereas Treg cells prevent excessive Th17 response and inflammation. Therefore, any factor that causes irregularity between these cells and their balance may induce embryo rejection. Overall, the immunological homeostasis is likely disturbed in URSA women and consequently the regular processes in the menstrual cycle are disorganized. The causes of disturbed homeostasis are not clear. Further studies are needed to clarify the factors that can affect immunological homeostasis in URSA women. 
